# Supplementary material for: Type I and III IFNs produced by the nasal epithelia and dimmed inflammation are features of alpacas resolving MERS-CoV infection
Source: PLoS Pathog. 2021 May 24;17(5):e1009229. doi: 10.1371/journal.ppat.1009229 (PMC8195365; doi:10.1371/journal.ppat.1009229)
Supplement: S1 Text — (DOCX) [file ppat.1009229.s001.docx]

**Animal study**

Fifteen 6-8 month-old alpacas (*Vicugna pacos*) were purchased by private sale and housed at the BSL-3 animal facilities of the Biocontainment Unit of IRTA-CReSA, in Barcelona, Spain. Animals were randomly numbered (AP1-AP15) and acclimated for one week. Twelve alpacas (AP1-AP12) were intranasally inoculated with a 10^7^ TCID_50_ dose of MERS-CoV Qatar15/2015 isolate in 3 mL saline solution (1.5 ml in each nostril) by using a mucosal atomization device (LMA MADgic, Teleflex Inc; USA), as described previously [1,2]. Three alpacas (AP13-AP15) were kept as non-infected controls.

All animals were monitored at least once per day for clinical signs (nasal discharge, coughing or dyspnea), food consumption, and rectal temperature until euthanasia. Nasal swab (NS) samples were collected on the day of euthanasia for MERS-CoV RNA detection and titration in Vero E6 cells as previously described [1]. Three alpacas were euthanized per day with an overdose of pentobarbital followed by exsanguination on 0, 1, 2, 3 and 4 days post-inoculation (dpi). Complete necropsies were performed, and the following tissues were collected: nasal turbinate, trachea, large and small bronchus, and lung parenchyma (apical, medial and caudal lobes). All tissues were collected in (a) Dulbecco's Modified Eagle Medium (DMEM) supplemented with 100 U/ml penicillin and 100 μg/ml streptomycin for virus isolation and genomic RNA detection; in (b) 10% neutral-buffered formalin for histopathology and immunohistochemistry (IHC); and in (c) methacarn (60% absolute methanol, 30% chloroform and 10% Glacial acetic acid) fixative for cytokine, MERS-CoV genomic and subgenomic mRNA detection in paraffin embedded tissues.

**Genomic viral RNA detection by RT-qPCR**

MERS-CoV viral RNA obtained from NS and tissue samples were prepared and analyzed according to a previously published RT-qPCR protocol [1]. Genomic MERS-CoV RNA was quantified by the UpE qPCR [3]. Samples with a cycle threshold of less than 40 were considered positive.

**Virus isolation from nasal swabs and tissues**

NS samples collected at various dpi and respiratory tract tissues obtained at 2 dpi were evaluated for the presence of infectious virus by titration in Vero E6 cells, as previously reported by scoring for the presence of CPE at 6 days after culturing [1]. The amount of infectious virus in each sample was calculated by determining the TCID50.

**Histopathology and Immunohistochemistry**

A monoclonal mouse anti-MERS-CoV N protein antibody (Sino Biological Inc., Beijing, China) was used to detect the presence of MERS-CoV antigen, following a previously established protocol [4]. A grading system for IHC was established by a board-certified veterinary pathologist (-, no positive cells detected; +/-, less than 10 positive cells per tissue section; +, 10 to 50 positive cells per tissue section; ++, 50 to 150 positive cells per tissue section; +++, 150 to 300 positive cells per tissue section; and ++++, more than 300 positive cells per tissue section). S1 Table summarizes the distribution and number of MERS-CoV infected cells found in the different tissue sections for each inoculated animal. DPP4 staining was performed in the alpaca lung by using a polyclonal goat IgG anti-human DPP4 antibody (R&D Systems, Abingdon, UK) following a previously published protocol [4].

**Methacarn-fixed paraffin-embedded tissue specimens**

For the assessment of cytokine mRNA profiles and presence of genomic and subgenomic MERS-CoV, nasal turbinates, trachea and lung were fixed by immersion in methacarn for the best preservation of RNA [5] and paraffin embedded. Tissue specimens were cut into four serial sections of 6 to 7 μm each and subsequently mounted onto Leica RNase-free PEN slides (Leica, Bannockburn, IL, USA). Prior to deparaffinization, the slides were air-dried for 30 min, followed by staining with 1% Cresyl Violet acetate for MFPE-nasal specimens and dehydrated through a series of graded ethanol steps prepared with RNase-free water before being air-dried. The whole process was performed in an RNase-free environment. MFPE-tracheal and lung samples were immediately processed for RNA isolation by scraping the whole section from the slides and referred thereafter to as ‘scraped’ tissues. MFPE-nasal specimens were processed for LCM prior to RNA extraction. Histopathology was also performed on tracheal and lung MFPE sections stained with hematoxylin and eosin.

**Primer design and validation of selected alpaca innate immune response and reference genes**

Alpaca genes and mRNAs [6] were obtained from the GenBank database (https://www.ncbi.nlm.nih.gov/genbank). Primers were designed through comparative genomics of sequences of alpaca (*Vicugna pacos*) and also other camelid species. Comparison of mRNA and genomic sequences of each studied gene were performed with the alignment tool ClustalW to determine exon boundaries, even in some instances exons were already annotated in camelid genomes. Primer pairs were designed with the Primer3 (http://bioinfo.ut.ee/primer3-0.4.0/), or Primer Express 2.0 (ThermoFisher Scientific, Life Technologies, Waltham, USA) with the following specifications: (i) bind at different exons or span exon-exon boundaries to avoid amplification of residual contaminating genomic DNA, (ii) 17-23 nucleotides in length, (iii) GC content between 45 and 55%, (iv) amplicon length of approximately 80-200 bp, (v) melting temperature (Tm) of primers between 57-63°C with less than 2°C difference within primer pairs, and (vi) avoiding primer hairpin, self-primer dimer or cross-primer dimer formation. Furthermore, the avoidance of primer secondary structure arrangement and the specificity of each primer sequence was assessed *in silico* through the Beacon Designer (http://www.premierbiosoft.com/qOligo/Oligo.jsp?PID=1), by selecting for primers with greater ΔG than - 3.5 kcal/mol when possible, and Blast (https://blast.ncbi.nlm.nih.gov/Blast.cgi), respectively. Potential transcription of predicted pseudogenes was discarded by carrying out promoter region analyses.

Stimulated PBMCs from healthy alpacas were used for primer validation. Phytohemagglutinin, a combination of phorbol 12-myristate 13-acetate and ionomycin calcium salt, or Poly(I:C)-LMW/LyoVec stimulated PBMCs were used to induce the expression of the genes of interest. Total RNA was isolated from PBMCs using the RNeasy Mini kit (Qiagen, Germany), which was reverse-transcribed into cDNA using Primescript First-strand (Takara, Japan). Primer efficiency, specificity, optimal annealing temperature, and differences of expression between stimulated and non-stimulated PBMCs were assessed by combining conventional PCR, gradient PCR, 2-step qPCR, Fluidigm Biomark qPCR and melting temperature analyses. Minus RT controls were included in all PCR assays to check the presence of DNA contamination. The whole set of reagents was fully validated prior to the study of the innate immune responses occurring in alpaca upon MERS-CoV infection.

**Fluidigm microfluidic quantitative PCR assay**

The 96.96 Dynamic Array IFC was used to analyze the expression of 40 genes (37 target genes and 3 reference genes) and detect genomic and subgenomic viral regions using the UpE and M mRNA assays, respectively. This assay was performed in MFPE tissues from micro-dissected (nasal epithelia and submucosa) or scraped (trachea and lung) tissue sections of MERS-CoV infected (AP1-12) and non-infected (AP13-15) alpacas at different dpi (1 to 4 dpi). Briefly, cDNA samples, prepared as above, were preamplified for 16 cycles using a Preamp Master Mix (Fluidigm Corporation, South San Francisco, USA), treated with Exonuclease I (New England Biolabs, Ipswich, USA), diluted 1:20 with Nuclease free H_2_O, loaded in duplicates into the corresponding array inlets and distributed across multiple reaction chambers. Quantification of the samples was performed on a Biomark HD system. The thermal cycle of the microfluidic qPCR was 60 s at 95°C, followed by 30 cycles of 5 s at 96°C and 60 s at 60°C. A dissociation step using EvaGreen detection was included for all reactions and was coupled with melting temperature analysis in order to confirm specific PCR amplification of the designed primers. Additionally, total RNA extracts from MERS-CoV infected nasal epithelia of AP5, 6, 7, 8, 9 and 11 were pooled at the same proportion per animal and used to generate cDNA controls for validation of gene expression assays. The pooled cDNA controls were serially diluted 1:4 (1:4, 1:16, 1:64, 1:256, 1:1024) and assayed in triplicates to create relative standard curves and calculate primer efficiencies (S3 Table). Non-template controls (NTC) without nucleic acids were also included in the assays.

**Data analyses and normalization of microfluidic qPCR assay**

Data from the samples used for gene expression analyses by microfluidic qPCR was collected using the Fluidigm Real-Time PCR analysis software 4.1.3 (Fluidigm Corporation, South San Francisco, USA) and analyzed using the DAG expression software 1.0.5.6 [7] to apply the relative standard curve method (see Applied Biosystems user bulletin #2). Briefly, the Cq threshold detection value was set at 0.020, the amplification quality threshold cut-off value was established at 0.65 and amplification specificity was assessed by Tm analyses for each reaction. All reactions quantified before the assay endpoint showed specific amplification. Cq values obtained at the Fluidigm qPCR from the serially diluted cDNA controls were used to create standard curves for each gene, and to extrapolate the quantity values of the studied samples. R-squared values were determined for each standard curve and the specific PCR efficiencies were calculated by applying the formula (10^(-1/slope value)-1)*100 (S3 Table). Multiple reference gene normalization was performed by using *GAPDH*, *HPRT1* and *UBC* as specific endogenous controls. After, their suitability for normalization procedures was confirmed by control-gene stability analyses using the DAG expression software 1.0.5.6 [7].

**Generation of graphs and heatmaps**

All the line and bar graphs were created with Prism version 8 software (GraphPad Software Inc., La Jolla, CA). Data obtained from the relative quantification analyses were plotted as heatmaps, using the Pheatmap package in R program (https://cran.r-project.org/).

**References**

1. Vergara-Alert J, van den Brand JMA, Widagdo W, Muñoz M, Raj VS, Schipper D, et al. Livestock susceptibility to infection with middle east respiratory syndrome coronavirus. Emerging Infectious Diseases. 2017. pp. 232–240. doi:10.3201/eid2302.161239

2. Rodon J, Okba NMA, Te N, van Dieren B, Bosch BJ, Bensaid A, et al. Blocking transmission of Middle East respiratory syndrome coronavirus (MERS-CoV) in llamas by vaccination with a recombinant spike protein. Emerg Microbes Infect. 2019;8: 1593–1603. doi:10.1080/22221751.2019.1685912

3. Corman VM, Eckerle I, Bleicker T, Zaki A, Landt O, Eschbach-Bludau M, et al. Detection of a novel human coronavirus by real-time reverse-transcription polymerase chain reaction. Eurosurveillance. 2012;17. doi:10.2807/ese.17.39.20285-en

4. Te N, Vergara-Alert J, Lehmbecker A, Pérez M, Haagmans BL, Baumgärtner W, et al. Co-localization of Middle East respiratory syndrome coronavirus (MERS-CoV) and dipeptidyl peptidase-4 in the respiratory tract and lymphoid tissues of pigs and llamas. Transboundary and Emerging Diseases. 2019. pp. 831–841. doi:10.1111/tbed.13092

5. Dotti I, Bonin S, Basili G, Nardon E, Balani A, Siracusano S, et al. Effects of Formalin, Methacarn, and FineFIX Fixatives on RNA Preservation. Diagnostic Mol Pathol. 2010;19: 112–122. doi:10.1097/PDM.0b013e3181b520f8

6. Wu H, Guang X, Al-Fageeh MB, Cao J, Pan S, Zhou H, et al. Camelid genomes reveal evolution and adaptation to desert environments. Nat Commun. 2014;5: 5188. doi:10.1038/ncomms6188

7. Ballester M, Cordón R, Folch JM. DAG Expression: High-Throughput Gene Expression Analysis of Real-Time PCR Data Using Standard Curves for Relative Quantification. Ma’ayan A, editor. PLoS One. 2013;8: e80385. doi:10.1371/journal.pone.0080385
