# Supplementary figures and images for: Type I and III IFNs produced by the nasal epithelia and dimmed inflammation are features of alpacas resolving MERS-CoV infection
Source: PLoS Pathog. 2021 May 24;17(5):e1009229. doi: 10.1371/journal.ppat.1009229 (PMC8195365; doi:10.1371/journal.ppat.1009229)

# A

Trachea

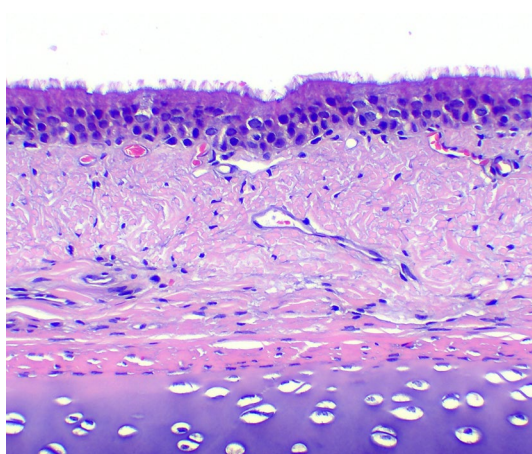

Bronchiole

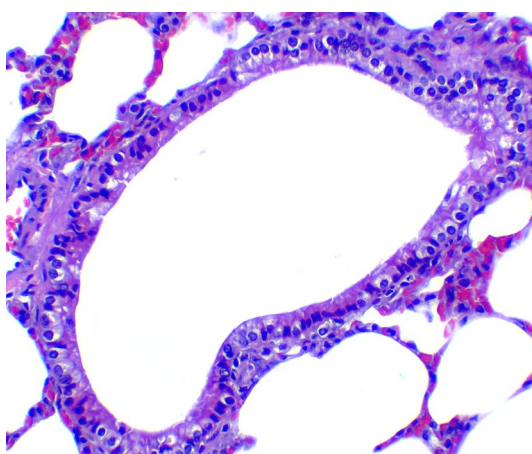

Large bronchus

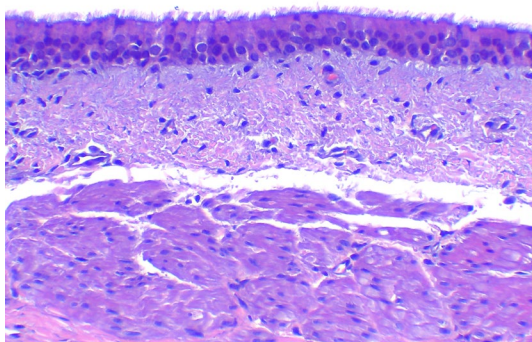

Alveoli

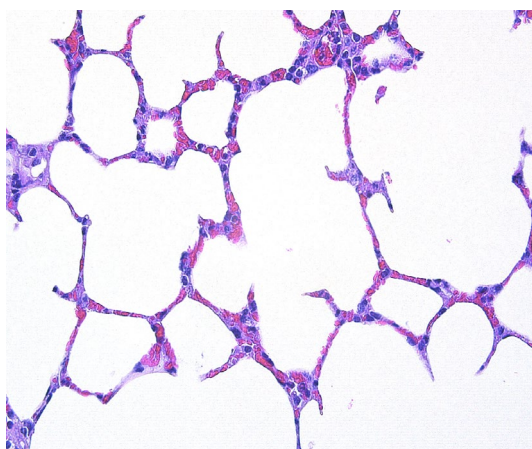

Small bronchus

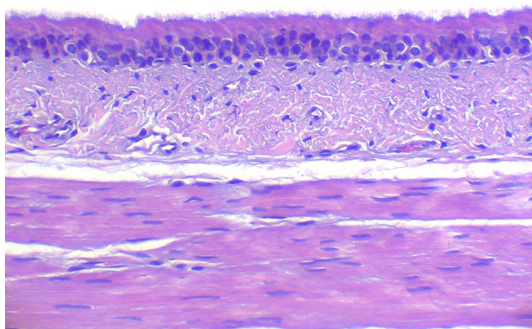

# B

DPP4

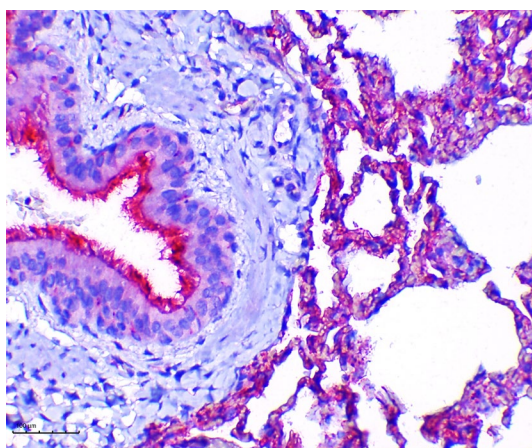

Negative control

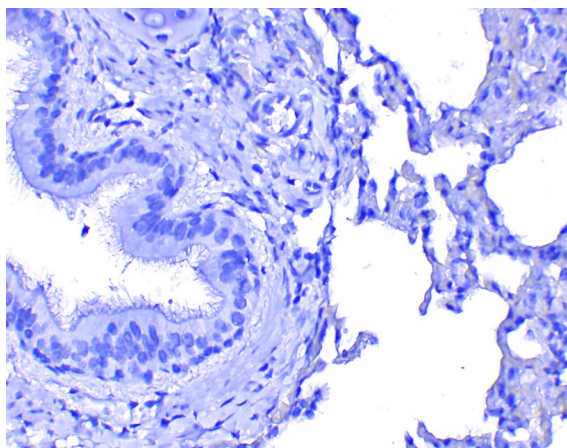

Supplement: S1 Fig — All respiratory tissues were fixed in 10% neutral-buffered formalin. (A) Tissue sections of trachea, bronchus and lung (bronchiole and alveoli) from non-infected control alpacas at 0 dpi stained with hematoxylin and eosin. (B) Pseudostratified columnar epithelial cells of bronchus and pneumocytes were positively stained for DPP4 (pink) by IHC. The negative control (without the primary anti DPP4 antibody) is shown at the right. Original magnification: ×400 for all tissues. (PDF) [file ppat.1009229.s002.pdf]

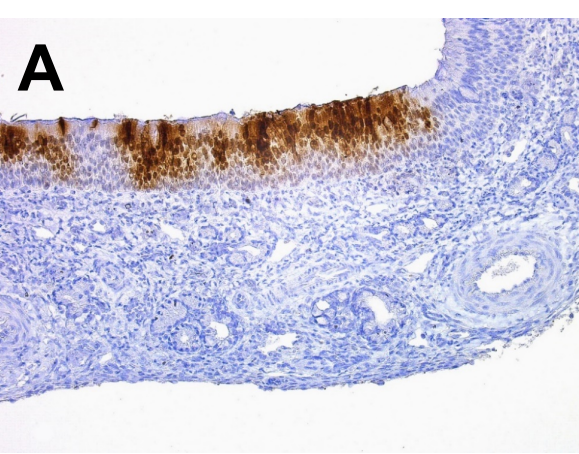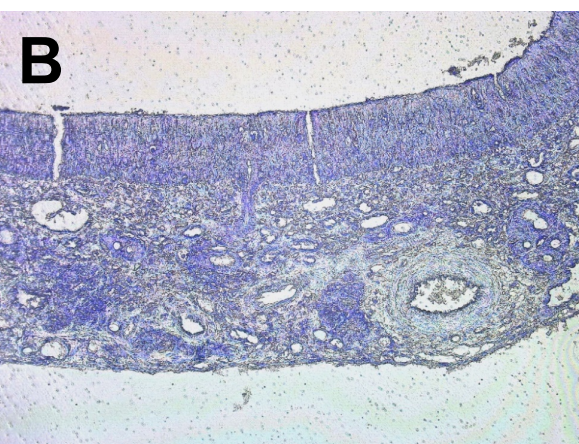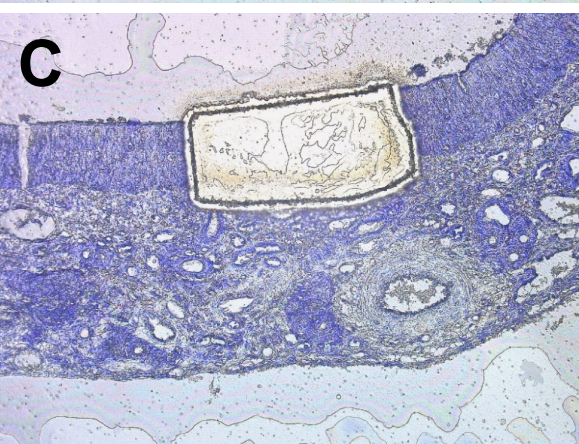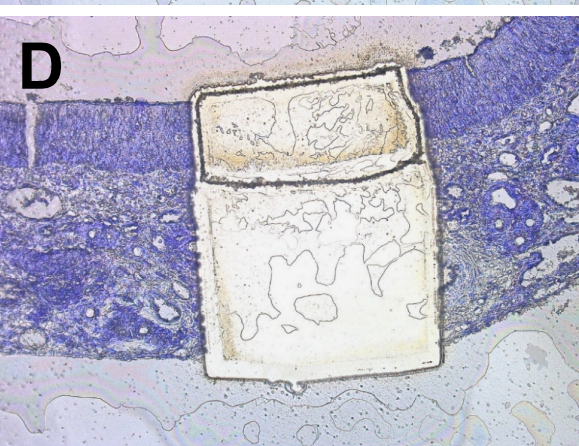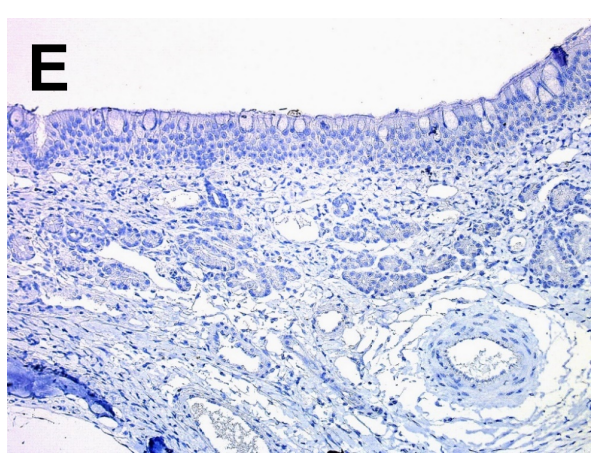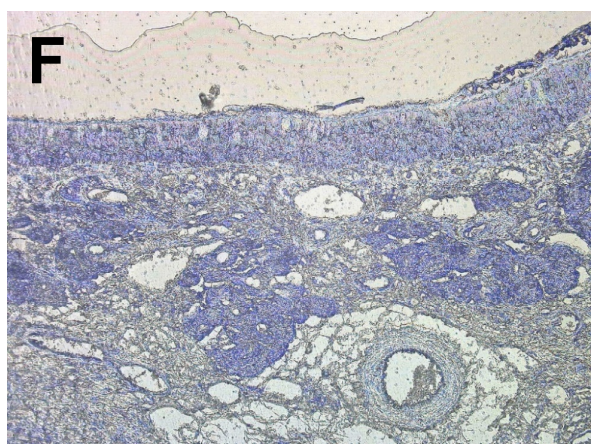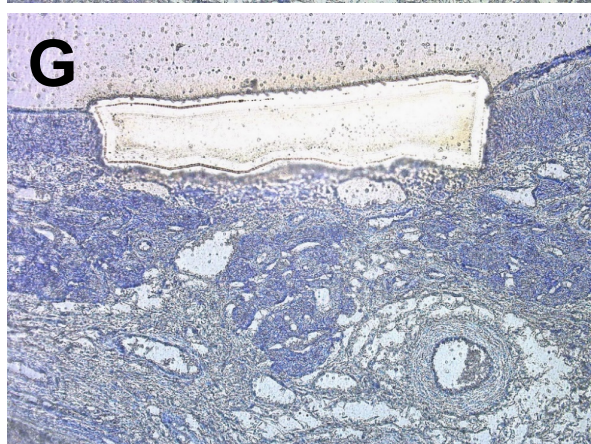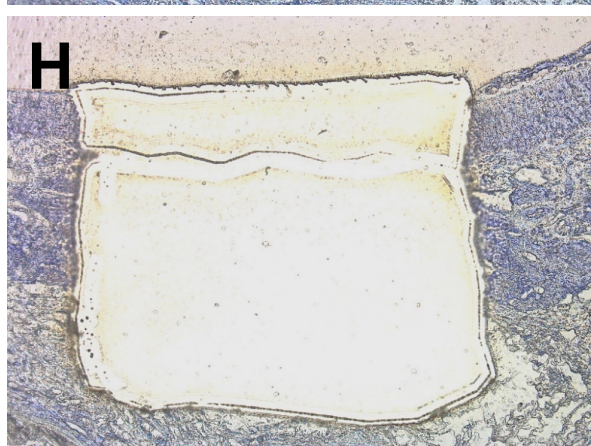

Supplement: S2 Fig — For each animal, four consecutive 6–7 μm sections from the same methacarn-fixed paraffin-embedded (MFPE) block were performed and mounted onto Leica RNase-free PEN slides. One of the sections was stained by IHC to detect the presence of the viral N protein and visualize heavily infected mucosa areas (A) from non-stained areas apparently devoided of virus (E). This IHC stained section served as a template to localize by overlapping on the other contiguous MFPE sections, stained only with cresyl violet, infected (B) and "non- infected" (F) areas prior LCM. Then LCM was applied to collect the respective selected areas in the mucosa (C and G) and the underlying submucosa (D and H). (PDF) [file ppat.1009229.s003.pdf]

**A**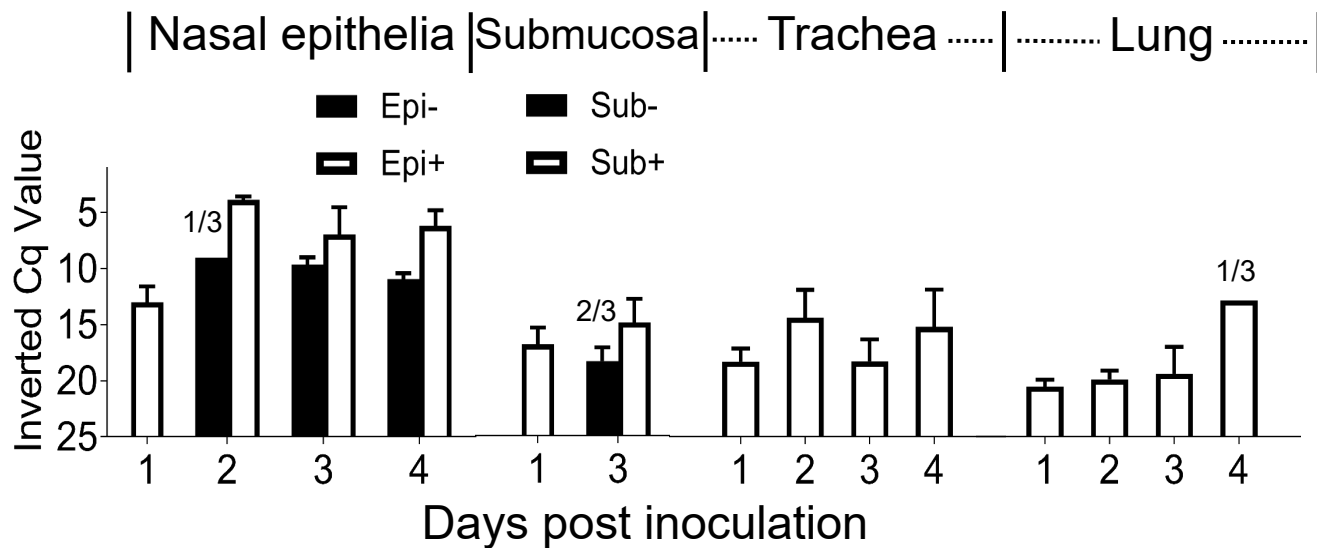**B**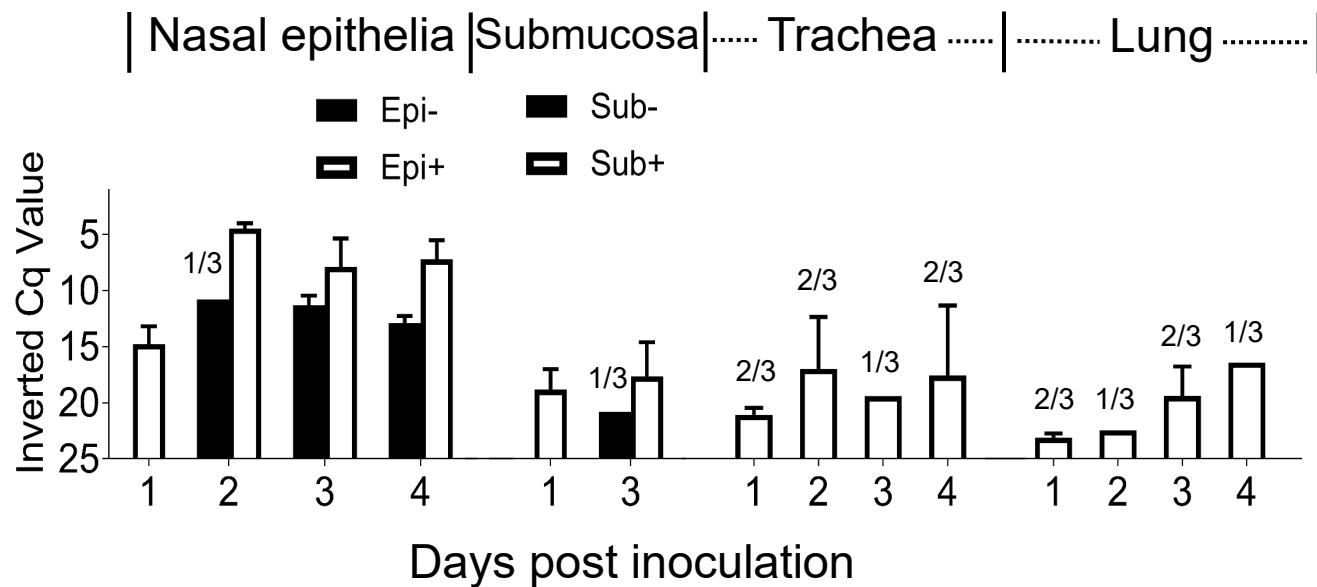

Supplement: S3 Fig — Micro- dissected (Nasal epithelia and underlying submucosa) and scrapped (Trachea and lung) MFPE tissue sections were prepared on the basis of an overlapping template section stained by IHC to localize the MERS-CoV N protein as described in S2 Fig. RNA extracted from these samples collected in alpacas prior (0 dpi, n = 3) MERS- CoV inoculation and after during 4 consecutive days (1 to 4 dpi, n = 3 per day) were converted into cDNA and (A) the MERS-CoV UpE gene and (B) the M mRNA amplified with a PCR microfluidic assay (Fluidigm Biomark). Error bars indicate SDs when results were positive in more than one animal. At 1 dpi, only Epi+ were sampled by LCM, the majority of cells were IHC negative. At 2 dpi, only one animal displayed distinct Epi- areas within the nasal turbinates which could be micro- dissected. All other animals at 2 dpi had a massive infection of nasal turbinates with few IHC negative cells. Sheet A in S2 Table provides detailed information on Cq values obtained for each animal and indicates also the samples with no detectable viral RNA in the nasal submucosa trachea and lung. Abbreviations: Epi-, non-infected nasal epithelia, as assessed by IHC; Epi+, MERS-CoV infected nasal epithelia, as assessed by IHC; Sub-submucosa underlying non-infected nasal epithelia, as assessed by IHC; Sub+, submucosa underlying MERS-CoV infected nasal epithelia, as assessed by IHC; MFPE, methacarn-fixed embedded-tissues; LCM, laser capture microdissection. (PDF) [file ppat.1009229.s004.pdf]

**A**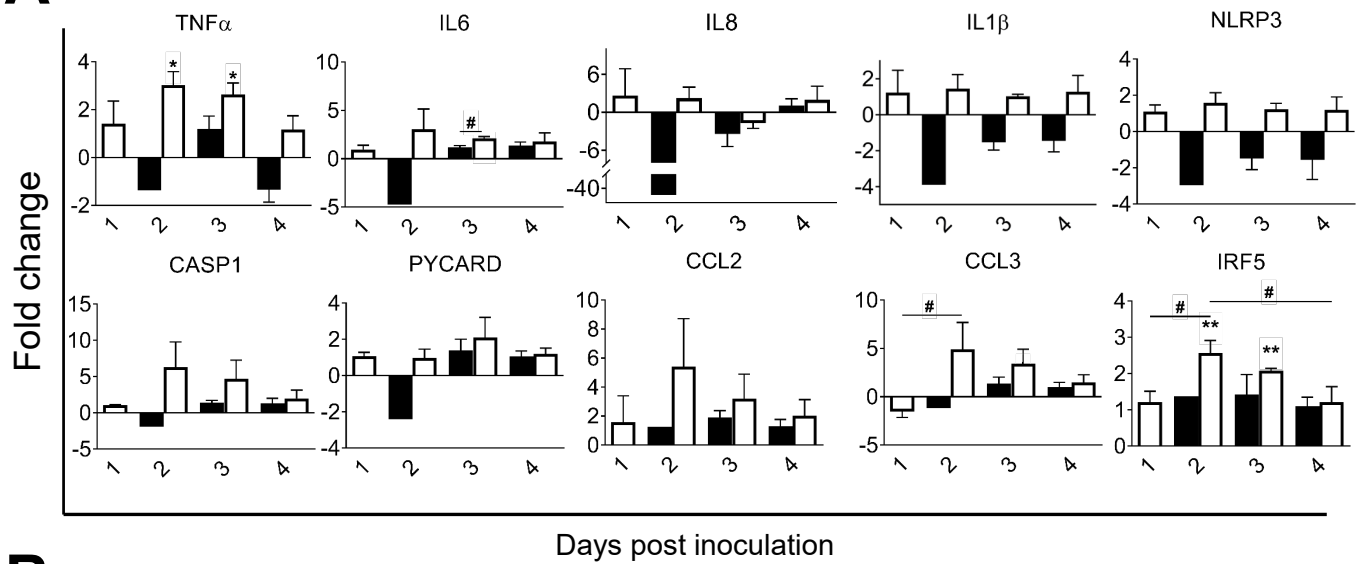**B**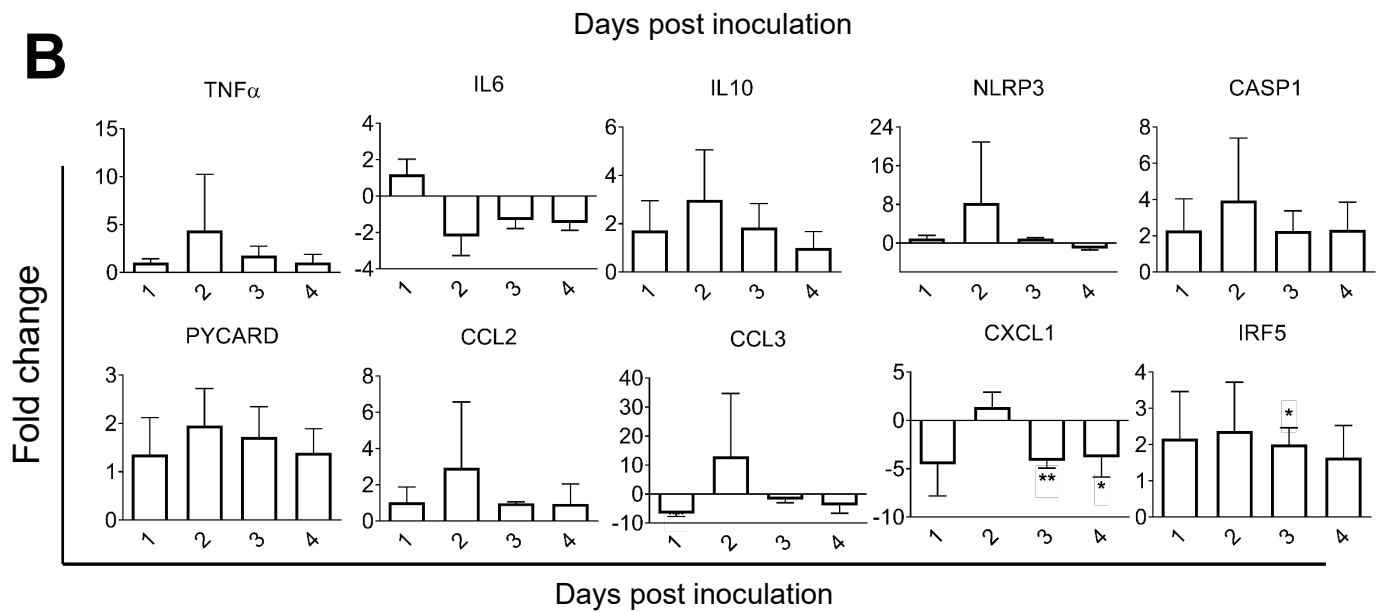**C**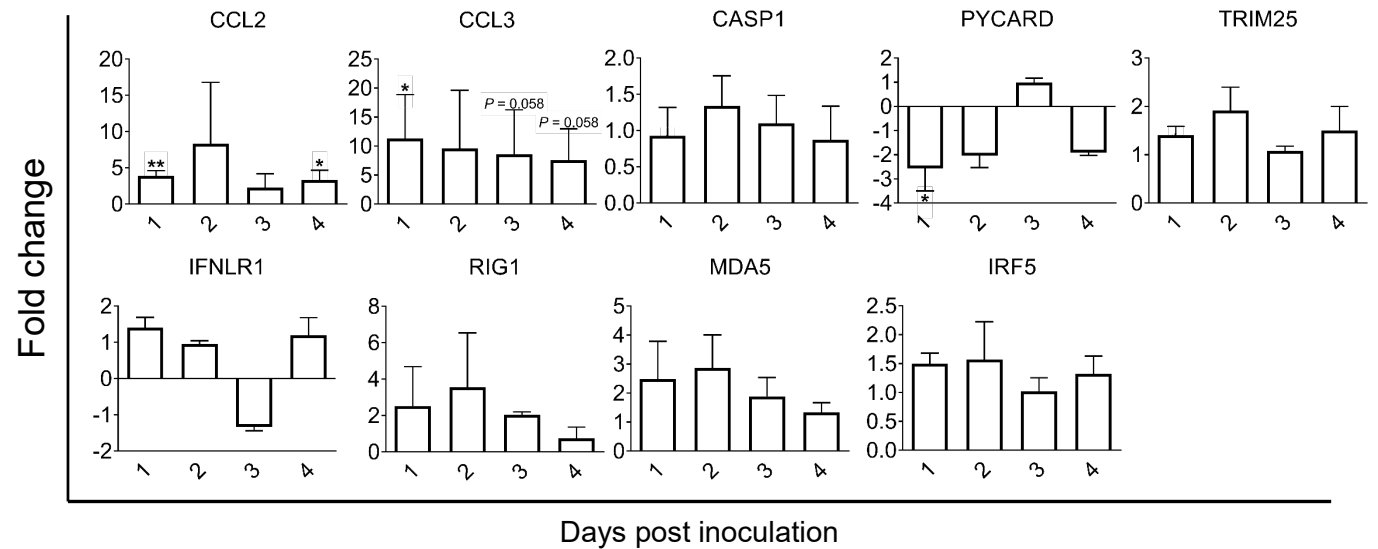

Supplement: S4 Fig — Micro-dissected nasal epithelia and scrapped (Trachea and lung) MFPE tissue sections were prepared on the basis of an overlapping template section stained by IHC to localize the MERS-CoV N protein as described in S2 Fig. RNA extracted from these samples collected in alpacas prior (0 dpi, n = 3) MERS-CoV inoculation and after during 4 consecutive days (1 to 4 dpi, n = 3 per day) were converted into cDNA. The Fluidigm Biomark microfluidic assay was used to amplify and quantify gene transcripts in (A) MERS-CoV positive nasal epithelia (white bars) and MERS-CoV negative nasal epithelia (black bars) as assessed by IHC (B), trachea and (C) lung at different dpi (1 to 4 dpi). At 1 dpi, only MERS-CoV positive nasal epithelia was sampled by LCM, the majority of cells were IHC negative. At 2 dpi, only one animal displayed distinct MERS-CoV negative areas within the nasal turbinates which could be micro-dissected. All other animals at 2 dpi had a massive infection of nasal turbinates with few IHC negative cells. Data are shown as means of Fc±SD. Statistical significance was determined by Student’s t- test. *P < 0.05; **P < 0.01; ***P < 0.001 (n = 3) when compared with the average values of non-infected alpacas (n = 3); #P < 0.05; ##P < 0.01; ###P < 0.001 (n = 3) when comparisons between groups are performed at different dpi. Significant upregulation of genes was considered if they met the criteria of a relative fold change of ≥2-fold with P < 0.05; similarly, significant downregulation of genes was considered if they met the criteria of a relative fold change of ≤2-fold with P < 0.05. (PDF) [file ppat.1009229.s005.pdf]

**A**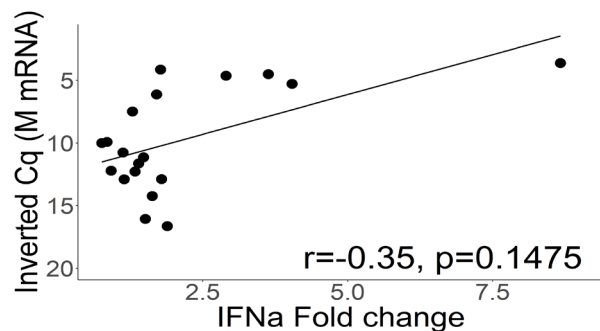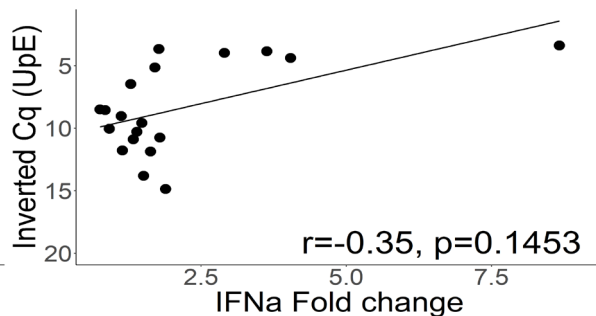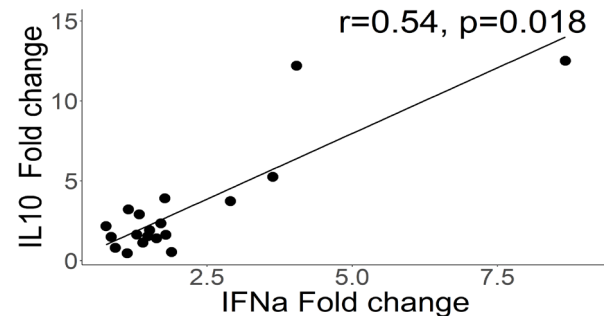**B**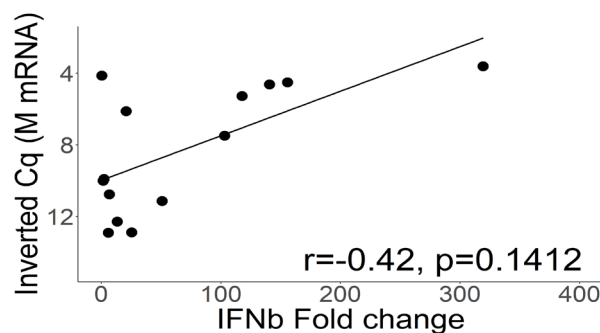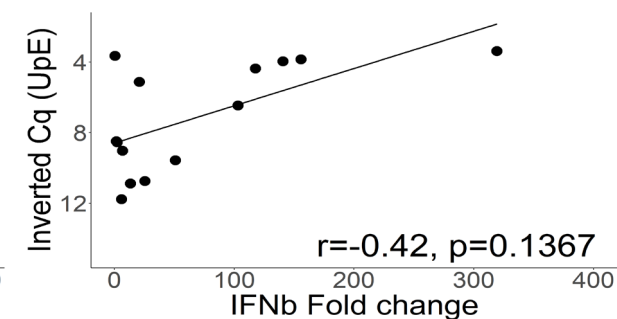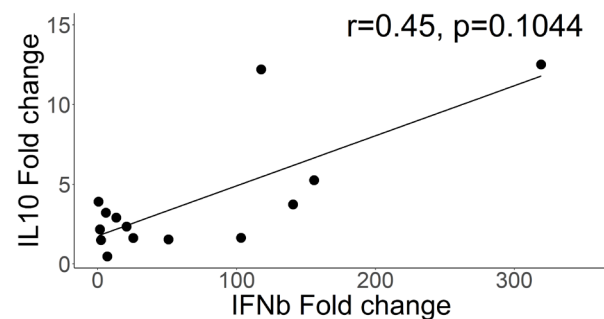**C**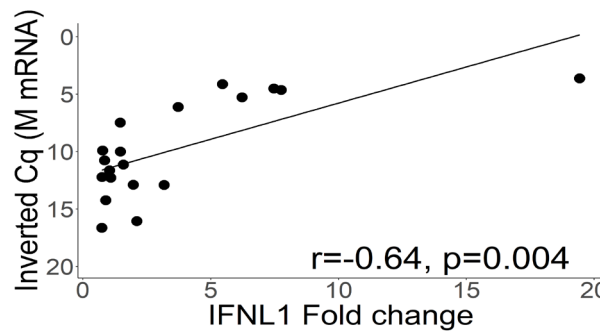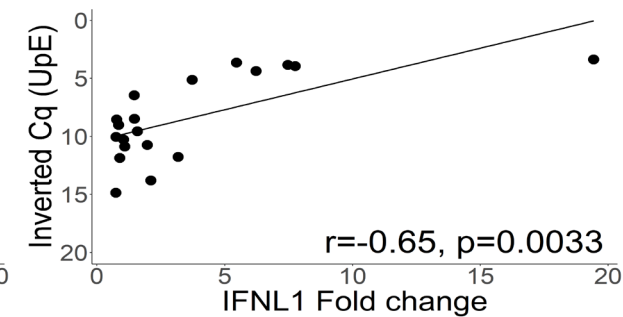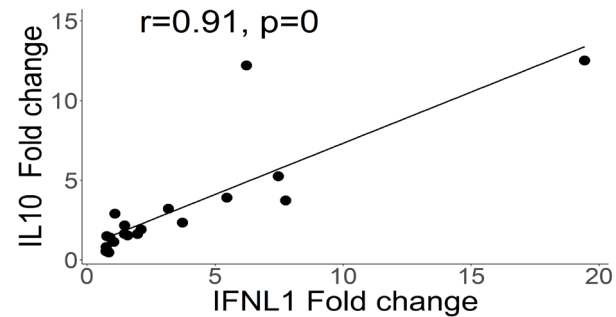**D**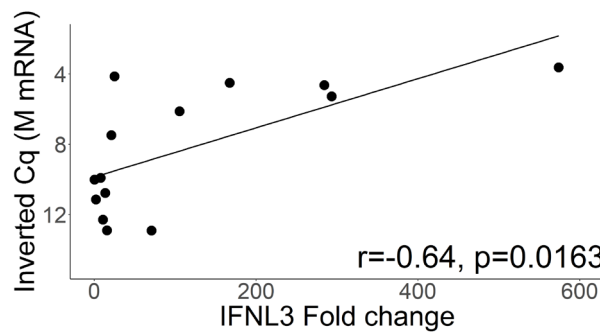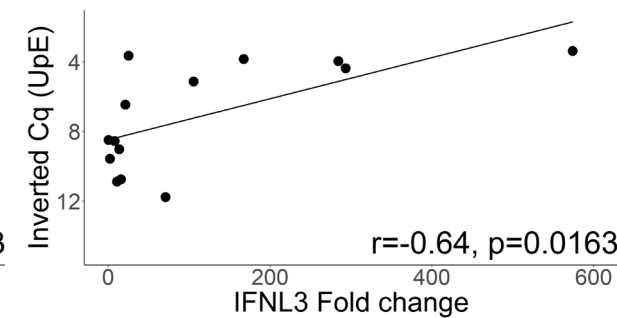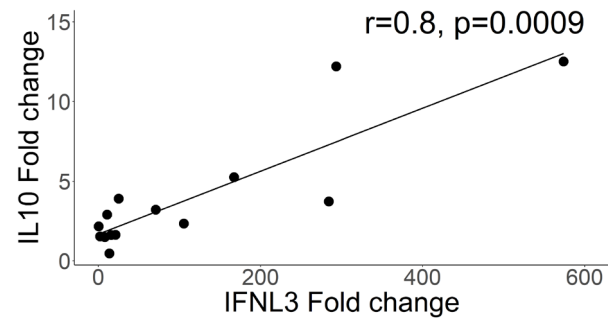

Supplement: S5 Fig — Portions of the nasal epithelia of each alpaca (AP1 to AP15) were micro-dissected from MFPE tissue sections on the basis of a section template stained by IHC allowing selection of areas positive or negative for the MERS-CoV N protein (S2 Fig). After RNA extraction and conversion to cDNA the Fluidigm Biomark microfluidic assay was used to amplify, among others, transcripts IFNα, IFNβ, IFNλ1, IFNλ3 and IL10 and the viral UpE gene and M mRNA from all nasal epithelia (negative and positive by IHC) collected at different dpi (0 to 4 dpi). Values are expressed, after normalization with the HPRT1, GAPDH and UBC gene transcripts, as fold changes of the expression of cytokines in infected animals versus control animals (AP13-15 sacrificed at 0 dpi) and inverted Cq for the viral UpE gene and M mRNA (Sheet A in S2 Table and S3 Fig). IFNβ (IFNb) was normalized with 1 dpi samples because it was not expressed at 0 dpi. Inverted Cq values of MERS-CoV M mRNA and UpE, and relative expression levels of IL10 were plotted against relative expression levels of (A) IFNα, (B) IFNβ, (C) IFNλ1 and (D) IFNλ3 in micro-dissected nasal epithelia. Correlation coefficients were established using the Spearman’s correlation test. dpi, days post inoculation. (PDF) [file ppat.1009229.s006.pdf]

**A**

AP13

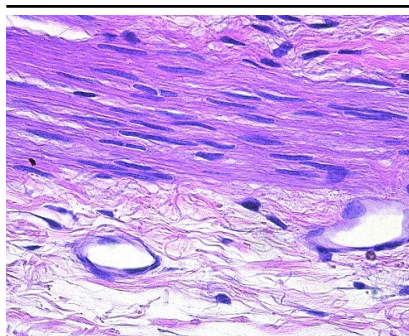

AP5

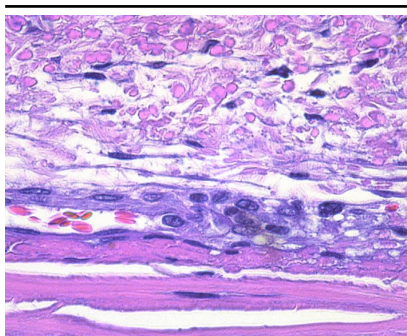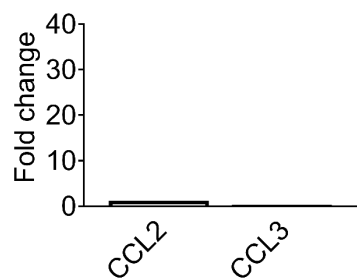**B**

CCL2

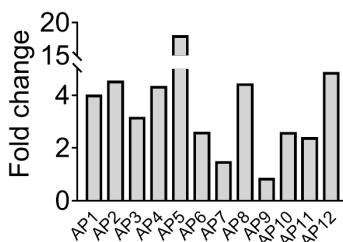

CCL3

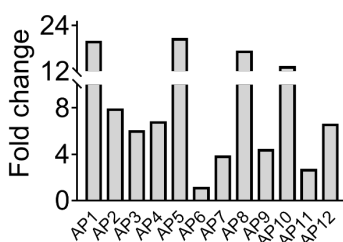

No. of inflammatory cells

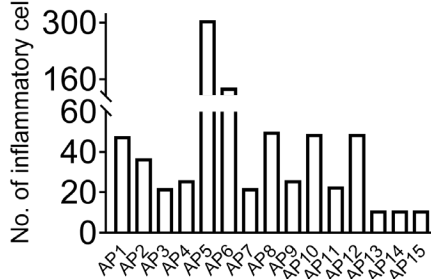**C**

AP2

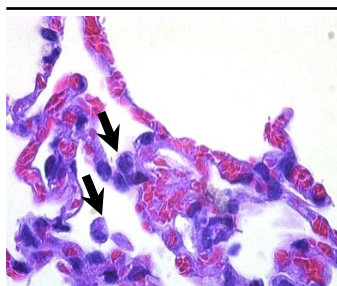

AP4

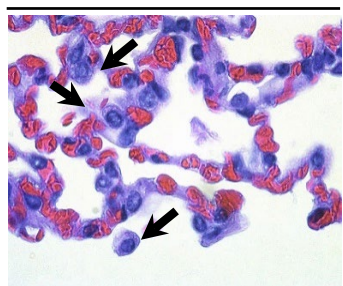

AP8

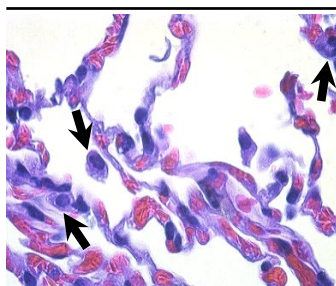

AP3

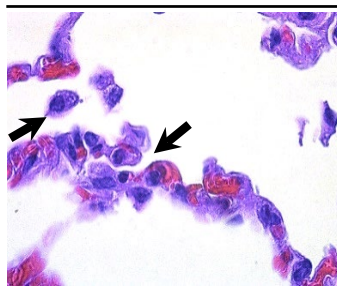

AP6

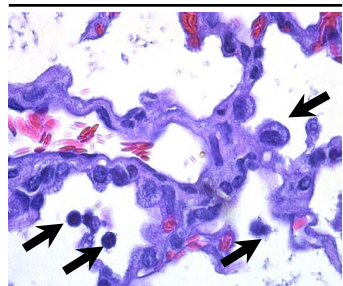

AP12

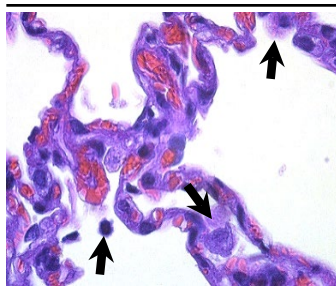**D**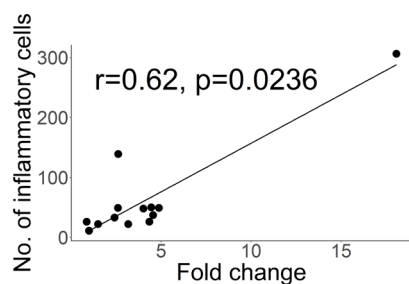**E**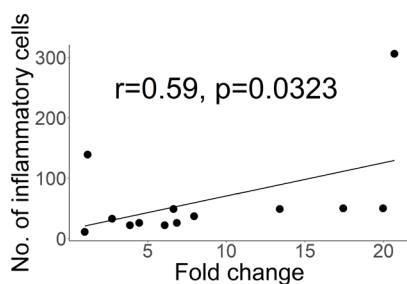

Supplement: S6 Fig — (A) No inflammatory cells nor CCL2 and CCL3 mRNA induction was seen in the MFPE tracheal submucosa of AP13 (0 dpi) and AP5 (2 dpi). (B) Relative expression of CCL2 and CCL3 mRNA was performed with a Fluidigm Biomark microfluidic assay in the lung of alpacas (including AP6). Inflammatory cells were counted in 3 microscopic fields (400X) per MFPE lung section in all animals, including non-infected controls. (C) The number of inflammatory cells in alveoli was high in AP6, lower in AP4, 8 and 12 (arrows), occasional in AP2 and 3 (arrow). Original magnification: ×1000 for all samples. Relative expression levels of (D) CCL2 and (E) CCL3 were plotted against the number of inflammatory cells in lungs. Correlation coefficients were established using the Spearman’s correlation. MFPE: methacarn-fixed paraffin-embedded. (PDF) [file ppat.1009229.s007.pdf]

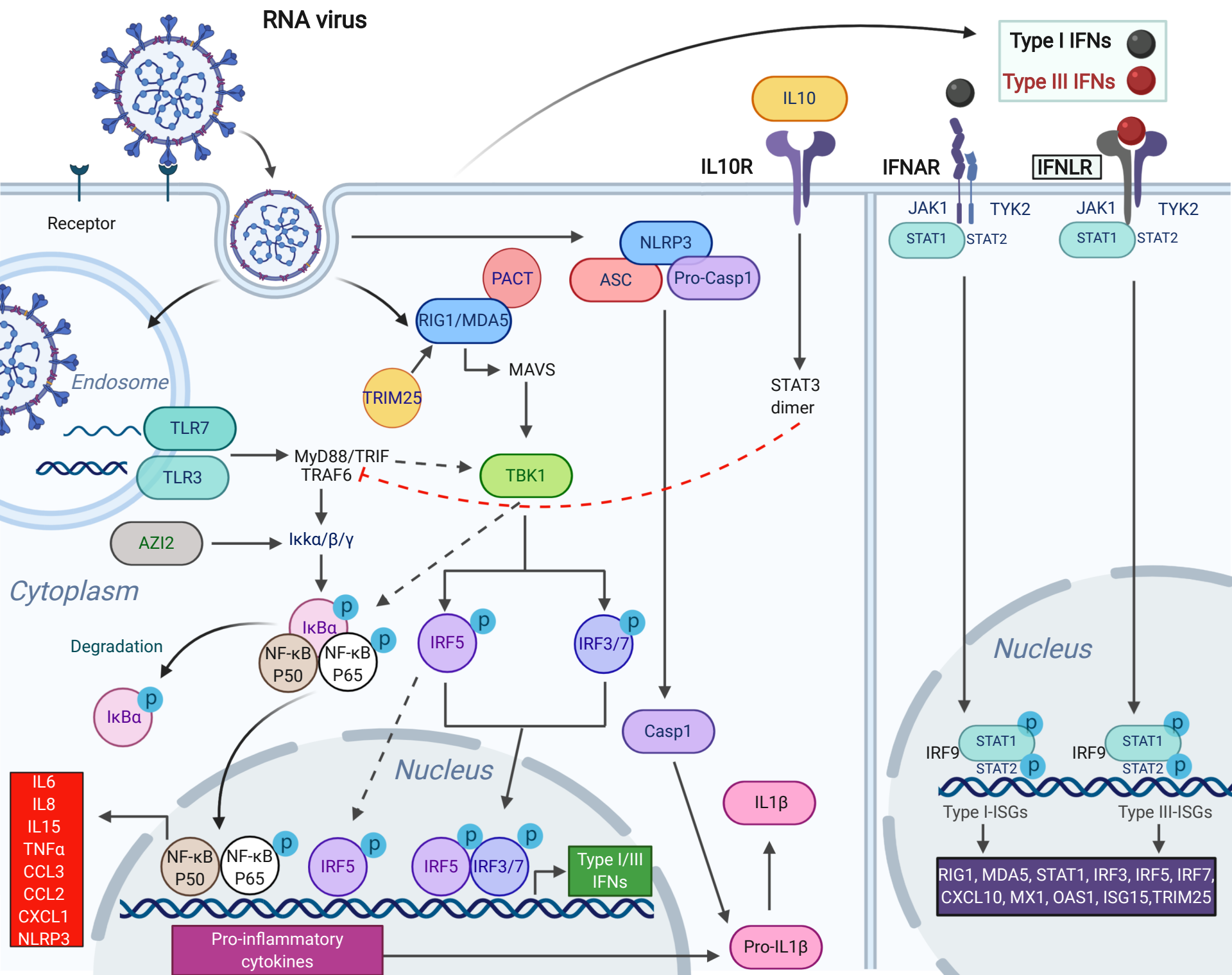

Supplement: S7 Fig — Upon sensing of RNA viruses, PRRs interact with their associated adapter proteins, thereby transmitting downstream signals to nuclear factor (NF-κB) and interferon regulatory factors (IRF3, IRF5 and IRF7) which further stimulate the production of pro-inflammatory cytokines (IL6, IL8, IL15, TNFα, CCL3, CCL2 CXCL1, NLRP3 and pro-IL1β) and IFNs (predominantly type I and III IFNs), respectively. Importantly, IRF5, is also a potent inductor of pro-inflammatory cytokines. The production of IFNs enhances the release of interferon stimulated genes (ISGs) which exert both, antiviral and host gene regulatory activities. IFNs can act in an autocrine and paracrine manner to induce the expression of ISGs via the JAK- STAT signaling pathway. While type I and III IFNs induce a similar set of ISGs (RIG1, MDA5, STAT1, IRF3, IRF5, IRF7, CXCL10, MX1, OAS1, ISG15, TRIM25), type I IFN signaling also activates pro-inflammatory cytokines and chemokines, which in turn recruit leukocytes to the site of infection. IL10 functions as an anti-inflammatory cytokine that activates STAT3 which is involved in the negative regulation of NF-κB. AZI2 and TBK1 (which also phosphorylate IRFs) participate positively in the NF-κB signaling pathway while IκBα inhibits translocation of NF-κB in the nucleus until degradation. Activation of the inflammasome, composed of NLRP3, PYCARD (ACS) and pro-CASP1 leads to the auto- cleavage of pro-CASP1 releasing CASP1 which further cleaves pro-IL1β into its mature form. The pro-apoptotic enzyme CASP10 and the adaptor CARD9 (acting downstream of C- type lectins signaling and upstream of the NF-κB pathway) are not represented in the diagram but were assayed for transcriptional regulation. Gene products, depicted with frames and colors were selected for microfluidic PCR assays, on basis of bibliographical data showing that they can be transcriptionally regulated following infection by RNA viruses. Furthermore, the selected genes sample most of the known signaling pathway [file ppat.1009229.s008.pdf]
