# Supplementary material for: Type I and III IFNs produced by the nasal epithelia and dimmed inflammation are features of alpacas resolving MERS-CoV infection
Source: PLoS Pathog. 2021 May 24;17(5):e1009229. doi: 10.1371/journal.ppat.1009229 (PMC8195365; doi:10.1371/journal.ppat.1009229)
Supplement: S1 Table — (DOCX) [file ppat.1009229.s009.docx]

**S1 Table. MERS-CoV N protein distribution in alpaca respiratory tracts by immunohistochemistry.**

|  | 1 dpi | | | 2 dpi | | | 3 dpi | | | 4 dpi | | |
| --- | --- | --- | --- | --- | --- | --- | --- | --- | --- | --- | --- | --- |
| **Tissue type** | AP1 | AP2 | AP3 | AP4 | AP5 | AP6 | AP7 | AP8 | AP9 | AP10 | AP11 | AP12 |
| Nasal turbinate | +/− | +/− | + | ++ | +++ | ++++ | ++ | + | + | + | + | + |
| Trachea | + | + | － | +/− | － | ++ | － | － | － | － | + | － |
| Large bronchus | － | － | － | ++ | + | ++ | － | － | － | － | + | － |
| Small bronchus | － | － | － | ++ | ­－ | － | － | － | － | － | ++ | － |
| Bronchiole in apical lung | － | － | － | － | + | + | － | － | － | － | － | + |
| Bronchiole in medial lung | － | － | － | － | + | － | － | － | － | + | － | － |
| Bronchiole in caudal lung | － | － | － | － | － | － | － | － | － | － | － | － |

Abbreviations: dpi, days post inoculation; AP, alpaca; -, no positive cells detected; +/-, less than 10 positive cells per tissue section; +, 10 to 50 positive cells per tissue section; ++, 50 to 150 positive cells per tissue section; +++, 150 to 300 positive cells per tissue section; and ++++, more than 300 positive cells per tissue section.
