# Supplementary material for: Type I and III IFNs produced by the nasal epithelia and dimmed inflammation are features of alpacas resolving MERS-CoV infection
Source: PLoS Pathog. 2021 May 24;17(5):e1009229. doi: 10.1371/journal.ppat.1009229 (PMC8195365; doi:10.1371/journal.ppat.1009229)
Supplement: S3 Table — (DOCX) [file ppat.1009229.s011.docx]

**S3 Table. Sequence and characteristics of primers used for microfluidic qPCR assays in alpacas.**

| **Gene category** | **Gene name** | **GenBank Accession number** | **Primer ID** | **Sequence (5' - 3')** | **Efficiency (%)** | **Coefficient of determination (R^2^)** |
| --- | --- | --- | --- | --- | --- | --- |
| Normalizer genes | GAPDH | XM_006210852 | GAPDH F | GGTCGGAGTGAACGGATTTGG | 99.60 | 0.997 |
|  |  |  | GAPDH R | TTGAGGTCAATGAAGGGGTCG |  |  |
|  | UbC | XM_031670203 | UbC F | AGGCGAAGATCCAAGACAAGG | 99.56 | 0.997 |
|  |  |  | UbC R | CCAAGTGCAGAGTGGATTCCT |  |  |
|  | HPRT1 | XM_031671409 | HPRT1 F | CAAAGATGGTCAAGGTCGCAA | 99.53 | 0.994 |
|  |  |  | HPRT1 R | TCAAATCCAACAAAGTCTGGTCT |  |  |
| Interferons | IFNα | XM_015242649 | IFNα F | TCTTCAGCGAGACACTTGCAA | 98.18 | 0.997 |
|  |  |  | IFNα R | GTTGGTCAGTGAGAATCATTTCCA |  |  |
|  | IFNβ | XM_006208258 | IFNβ F2 | GCATCCTCCAAATCGCTCTCC | 102.27 | 0.968 |
|  |  |  | IFNβ R2 | ATGCCAAGTTGCTGCTCCTTT |  |  |
|  | IFNλ1 | XM_006206664 | IFNλ1 F | CTGCCACATGGGCTGGTT | 100.49 | 0.945 |
|  |  |  | IFNλ1 R | CGATTCTTCCAAGGCATCCTT |  |  |
|  | IFNλ3 | XM_006219677 | IFNλ3 F | CCACCTGGCCCAATTCAA | 100.92 | 0.996 |
|  |  |  | IFNλ3 R | AGTGACTCTTCAAAGGCGTCCTT |  |  |
| Pattern recognition receptors | RIG1 (DDX58) | XM_031676595 | RIG1 F | ACAAGTCAGAACACAGGAATGA | 97.96 | 0.998 |
|  |  |  | RIG1 R | CTCTTCCTCTGCCTCTGGTTT |  |  |
|  | MDA5 (IFIH1) | XM_006196223 | MDA5 F | ACACCAGAGTTCAAGAGACTGTAT | 98.39 | 0.998 |
|  |  |  | MDA5 R | CACCATCATCGTTCCCCAAGA |  |  |
|  | TLR3 | XM_015249164 | TLR3 F | AGAAATAGACAGACAGCCAGAG | 95.66 | 0.999 |
|  |  |  | TLR3 R | TGCTCCTTTTGATGCTATTAACGA |  |  |
|  | TLR7 | XM_006212620 | TLR7 F | AGAGAGGAGTCACCAGCGTAT | 100.60 | 0.988 |
|  |  |  | TLR7 R | GACACAAATGCAAATGGAGAC |  |  |
|  | NLRP3 | XM_031673306 | NLRP3 F | ATGGCCACATGGATTTTTGC | 103.01 | 0.991 |
|  |  |  | NLRP3 R | AAACATTGGCATTGTCCCATTC |  |  |
| Transcription factors | STAT1 | XM_031678037 | STAT1 F | TCTCTGTGTCTGAAGTTCACCCT | 89.88 | 1.000 |
|  |  |  | STAT1 R | GGGAATCACAGGTGGGAAGGA |  |  |
|  | IRF3 | XM_006208451 | IRF3 F | TCACCACGCTACACCCTCTGGT | 98.60 | 0.989 |
|  |  |  | IRF3 R | GAGGCACATGGGCACAACCTTGA |  |  |
|  | IRF5 | XM_006202276 | IRF5 F | TCAGAAGGGCCAGACCAACACC | 98.59 | 0.995 |
|  |  |  | IRF5 R | TGCTACGGGCACCACCTGTA |  |  |
|  | IRF7 | XM_015251986 | IRF7 F | CGTGATGTTGCAAGACAACTCA | 99.88 | 0.998 |
|  |  |  | IRF7 R | TGGTTAACGCCTGGGTCTCT |  |  |
|  | NFKB1 | XM_031690344 | NFKB1 F | GGGACAGTGTCTTACACTTAGCAATC | 98.88 | 0.995 |
|  |  |  | NFKB1 R | CATCAGAAATCAAGCCAGATGTG |  |  |
| Interferon stimulated genes | CXCL10 (IP10) | XM_006198241 | CXCL10 F | CGTGTTGAGATTATTGCCACAATG | 95.77 | 0.997 |
|  |  |  | CXCL10 R | GAGGTAGCTTCTCTCTGGTCCT |  |  |
|  | MX1 | XM_006204960 | MX1 F | GAAGATGGTTTATTCTGACTCG | 100.39 | 0.999 |
|  |  |  | MX1 R | TTCTCCTCGTACTGGCTGT |  |  |
|  | OAS1 | XM_031670190 | OAS1 F | TGAAGAAGCAGCTCGGGAAAC | 96.77 | 0.997 |
|  |  |  | OAS1 R | AGTAACTGTCTTTTCTGGGCAGC |  |  |
|  | ISG15 | XM_015237784 | ISG15 F | CACAGCCATGGGTGGAATCCTG | 102.34 | 0.998 |
|  |  |  | ISG15 R | ACAGCATGGAGTCCCTCAGAGTC |  |  |
| Inflammatory cytokines and chemokines | IL10 | XM_006215461 | IL10 F | CTGCTGGAGGACTTTAAGGGT | 101.65 | 0.972 |
|  |  |  | IL10 R | AGGGGAGAAATCGATGACAGC |  |  |
|  | IL1β | XM_006203828 | IL1β F | AGGATATGAGCCGAGAAGTGGT | 101.17 | 0.982 |
|  |  |  | IL1β R | CCCTTTCATCACACAAGACAGGT |  |  |
|  | IL6 | XM_006201793 | IL6 F | TCTGGGTTCAATCAGGAGACCT | 100.70 | 0.986 |
|  |  |  | IL6 R | AGGGGTGCTTACTTCTTCTGGT |  |  |
|  | IL8 (CXCL8) | XM_006212530 | IL8 F | TGTGTGAAGCTGCAGTTCTGT | 94.73 | 0.995 |
|  |  |  | IL8 R | GCAGACCTCTCTTCCATTGGC |  |  |
|  | IL15 | XM_015249496 | IL15 F | CAGCCTACAGAAGGTCATGAAGTACTC | 94.09 | 0.997 |
|  |  |  | IL15 R | GGGTAACTCCTTAAGTATCGAAGAAGAG |  |  |
|  | TNFα | XM_006215316 | TNFα F | TGGCCCAGACCCTCAGATCA | 100.50 | 0.990 |
|  |  |  | TNFα R | TTCCAGCTTCACACCATTGGC |  |  |
|  | CCL3 (MIP1α) | XM_006213334 | CCL3 F | GCTCAGCGTCATGCAGGTGCC | 99.86 | 0.988 |
|  |  |  | CCL3 R | AGCAGGCGGTTGGGGTGTCAG |  |  |
|  | CCL2 (MCP1) | XM_006212021 | CCL2 F | CCAGTAAGAAGATCCCCATGCA | 99.63 | 0.997 |
|  |  |  | CCL2 R | GTGTGGTCTTGAAGATCACAGCTT |  |  |
|  | CXCL1 | XM_031684028 | CXCL1 F | CGTGCAGGGAATTCACTTCAA | 99.81 | 0.998 |
|  |  |  | CXCL1 R | GAGAGTGGCTACGACTTCCGTTT |  |  |
| Enzymes | CASP1 | XM_015249739 | CASP1 F | ACTCCACCAAGACCTCAACCAGT | 97.03 | 0.992 |
|  |  |  | CASP1 R | GGGTAAATCTCCGCTGACTTCTCG |  |  |
|  | CASP10 | XM_006205263 | CASP10 F | CGGTAGCCACGGGAACTGAGTCAT | 95.48 | 0.994 |
|  |  |  | CASP10 R | ATCTTGCCAGGACCCCTCCGAT |  |  |
|  | AZI2 (NAP1) | XM_006200749 | AZI2 F | TGAGCGTCTCCAGCGCTAA | 99.78 | 0.996 |
|  |  |  | AZI2 R | CTGCACTTGCGTCACCAGAT |  |  |
|  | PACT (PRKRA) | XM_006210217 | PACT F | TGCAGTTCCTGACCCCTTAATG | 99.10 | 0.995 |
|  |  |  | PACT R | GATGAATAGCCAGTTCCTGTAGTGAA |  |  |
|  | TBK1 | XM_031683111 | TBK1 F | GTACAGAAAGCAGAAAATGGACCAA | 99.69 | 0.997 |
|  |  |  | TBK1 R | AACTTGAAGGCCCCGAGAAA |  |  |
|  | TRIM25 | XM_031685141 | TRIM25 F | GCCCGAGCTCCTACAGTATGC | 99.83 | 0.997 |
|  |  |  | TRIM25 R | GAAGCGACGGTGTAGGTCTTG |  |  |
| Adaptors | NFKBIA (IκBα) | XM_031678782 | NFKBIA F | TCCCTCTTTTCCCCGCAGGTT | 99.00 | 0.996 |
|  |  |  | NFKBIA R | TGGAGTGGAGTCTGCTGCAGGT |  |  |
|  | CARD9 | XM_006218359 | CARD9 F | GGCAGTGCAAGGTCCTGAAC | 94.72 | 0.981 |
|  |  |  | CARD9 R | CAGGAGCACACCCACTTTCC |  |  |
|  | PYCARD (ASC) | XM_015236916 | PYCARD F | CAAGCCAGCACCGCACTT | 99.03 | 0.995 |
|  |  |  | PYCARD R | TCTGTCAGGACCTTCCCATACA |  |  |
| Receptors | IFNLR1 | XM_031683805 | IFNLR1 F | CAGGGTGTGTGATCTGGAAGAG | 100.99 | 0.996 |
|  |  |  | IFNLR1 R | GTCTGTGTCCAGAGAAATCCAGG |  |  |

Selected normalizer and immune response genes from alpacas were identified in the GenBank (accession number provided) and forward (F) and reverse primers (R) designed. Pair of primers efficiency and coefficients of determination were calculated as indicated in material and methods and supplementary material and methods.
